# NaCl-Assisted Chemical Vapor Deposition of Large-Domain Bilayer MoS_2_ on Soda-Lime Glass

**DOI:** 10.3390/nano12172913

**Published:** 2022-08-24

**Authors:** Qingguo Gao, Lvcheng Chen, Simin Chen, Zhi Zhang, Jianjun Yang, Xinjian Pan, Zichuan Yi, Liming Liu, Feng Chi, Ping Liu, Chongfu Zhang

**Affiliations:** 1 School of Electronic Information, University of Electronic Science and Technology of China, Zhongshan Institute, Zhongshan 528402, China; 2 School of Information and Communication Engineering, University of Electronic Science and Technology of China, Chengdu 611731, China

**Keywords:** bilayer MoS_2_, chemical vapor deposition, NaCl, glass

## Abstract

In recent years, two-dimensional molybdenum disulfide (MoS_2_) has attracted extensive attention in the application field of next-generation electronics. Compared with single-layer MoS_2_, bilayer MoS_2_ has higher carrier mobility and has more promising applications for future novel electronic devices. Nevertheless, the large-scale low-cost synthesis of high-quality bilayer MoS_2_ still has much room for exploration, requiring further research. In this study, bilayer MoS_2_ crystals grown on soda-lime glass substrate by sodium chloride (NaCl)-assisted chemical vapor deposition (CVD) were reported, the growth mechanism of NaCl in CVD of bilayer MoS_2_ was analyzed, and the effects of molybdenum trioxide (Mo) mass and growth pressure on the growth of bilayer MoS_2_ under the assistance of NaCl were further explored. Through characterization with an optical microscope, atomic force microscopy and Raman analyzer, the domain size of bilayer MoS_2_ prepared by NaCl-assisted CVD was shown to reach 214 μm, which is a 4.2X improvement of the domain size of bilayer MoS_2_ prepared without NaCl-assisted CVD. Moreover, the bilayer structure accounted for about 85%, which is a 2.1X improvement of bilayer MoS_2_ prepared without NaCl-assisted CVD. This study provides a meaningful method for the growth of high-quality bilayer MoS_2_, and promotes the large-scale and low-cost applications of CVD MoS_2_.

## 1. Introduction

In recent years, with the rapid development of microelectronic devices, microelectronic technology based on silicon materials has encountered some bottlenecks, such as high power consumption, size reduction and slow performance improvement. New technology and material research is the key factor that is expected to break through the current technological bottlenecks [1,2,3]. Therefore, two-dimensional transition metal dichalcogenides (TMDs) have attracted extensive research attention due to their unique electronic and optoelectronic properties [4,5,6]. Among these two-dimensional TMDs, molybdenum disulfide (MoS_2_) has received the most extensive research attention [7,8,9,10,11]. Compared with graphene, MoS_2_ has greater advantages in transistor application because of its band gap, high switching ratio and strong inhibition of the short-channel effect [12,13]. Compared with silicon transistors, MoS_2_ transistors present lower power consumption and better inhibition of the short-channel effect [9,14]. For instance, based on side-wall MoS_2_ transistors, the physical channel length of transistors can reach 0.34 nm [9]. 

To realize the industry-compatible integration of these applications, the batch production of high-quality and large-crystal MoS_2_ films at low cost is of great importance. In the early stages of MoS_2_ research, diverse preparation methods were reported [15]. Xia et al. synthesized MoS_2_ crystals through a hydrothermal reaction followed by low-temperature crystallization annealing [16]. Eda et al. obtained large-scale few-layer MoS_2_ by ultrasound-assisted lithium intercalation exfoliation [17]. However, the layer number and size of MoS_2_ prepared by the above methods are difficult to control. Lou et al. used vapor-phase vulcanization to deposit a nano-scale Mo source on SiO_2_ sheets for sulphuration, and obtained continuous few-layer MoS_2_ films [18]. However, this method is very likely to cause a decrease in crystallinity, thereby leading to reduced mobility [19]. In the research on MoS_2_, chemical vapor deposition (CVD) is generally used over other methods. Wang et al. successfully prepared MoS_2_ crystals by CVD using sulfur (S) powder and molybdenum trioxide (MoO_3_) as precursors and SiO_2_/Si as a growth substrate [20], with the size reaching micrometers [21,22,23,24]. So far, the domain size of monolayer MoS_2_ crystal growth based on CVD has reached the millimeter level [25,26].

As we all know, in the CVD synthesis of MoS_2_, substrates play a critical role [27]. Moreover, in recent years, various substrates have been used in the growth of CVD MoS_2_ by different research groups [28,29,30,31]. Yang et al. synthesized highly oriented centimeter-scale MoS_2_ single crystals using gold foil as the growth substrate [31]. Li et al. prepared wafer-scale MoS_2_ single crystals on sapphire substrates through step-induced nucleation [10,32]. However, the triangular domain size of these MoS_2_ crystals is only tens of microns, and the cost of the sapphire and gold foil substrates is relatively high. In recent years, soda-lime glass has also received extensive attention in the research on growing MoS_2_ through CVD due to its advantages such as low cost, high growth rate, and large domain size [33,34,35,36]. As the growth substrate for MoS_2_, soda-lime glass has high fluidity under a high-temperature melting state, and can weaken lattice mismatch during the adsorption–diffusion–nucleation of precursor [4,27]. Additionally, Na+ released during melting can reduce the reaction barrier of MoS_2_ and promote its growth [37]. Chen et al. successfully grew high-quality millimeter-sized single-layer MoS_2_ on the molten soda-lime glass substrate through atmospheric pressure CVD [38], demonstrating the great advantages of the soda-lime glass substrate. 

Compared with single-layer MoS_2_, bilayer MoS_2_ has a higher density of states and electron mobility due to its smaller indirect band gap, and retains the characteristics of atomic layer thickness [39], contributing to its higher potential in the field of novel electronic devices [11,13,40]. However, the large-scale growth of high-quality bilayer MoS_2_ on low-cost glass substrates still needs further research. For example, the domain size of CVD bilayer MoS_2_ single crystals is still about 200 μm [13,41], which is significantly smaller than the crystal size of monolayer MoS_2_. Recently, some research teams have tried to use some substances with a catalytic effect to assist the growth of MoS_2_, and achieved improved results [42,43,44,45]. Through the use of NaCl and Mo foil used as Mo source, Yang et al. successfully grew 100 μm high-quality bilayer MoS_2_ domains on soda-lime glass substrates by face-to-face CVD [46]. Nevertheless, this work focused on the synthesis of large-scale continuous MoS_2_ films, and the domain size of the bilayer MoS_2_ could be further improved by the optimization of growth conditions. Moreover, it has been shown that the growth rate of MoS_2_ from Mo in a 0-valence state is not as efficient as that of Mo in a 6-valence state in MoO_3_ [47]. However, nowadays, there is a lack of relevant research on directly introducing NaCl into MoO_3_ to assist high-quality bilayer MoS_2_ growth on low-cost soda-lime glass substrates.

In this work, we reported the results of CVD-based growth of bilayer MoS_2_ on melt soda-lime glass by introducing NaCl into the Mo source (MoO_3_). It was found that the introduction of NaCl could promote the growth of bilayer MoS_2_. Additionally, under NaCl-assisted growth, the synthesis of bilayer MoS_2_ crystals with different Mo masses and growth pressures was explored. Finally, by optimizing the process parameters, the size of bilayer MoS_2_ crystals was increased from the initial 50 μm to 214 μm, and the proportion of bilayer structure increased from the initial 40% to 85%. This study provides a valuable approach for the growth of bilayer MoS_2_ crystals, and is meaningful for the application of TMDs in future high-performance electronics.

## 2. Experiments and Methods

**Figure 1:**
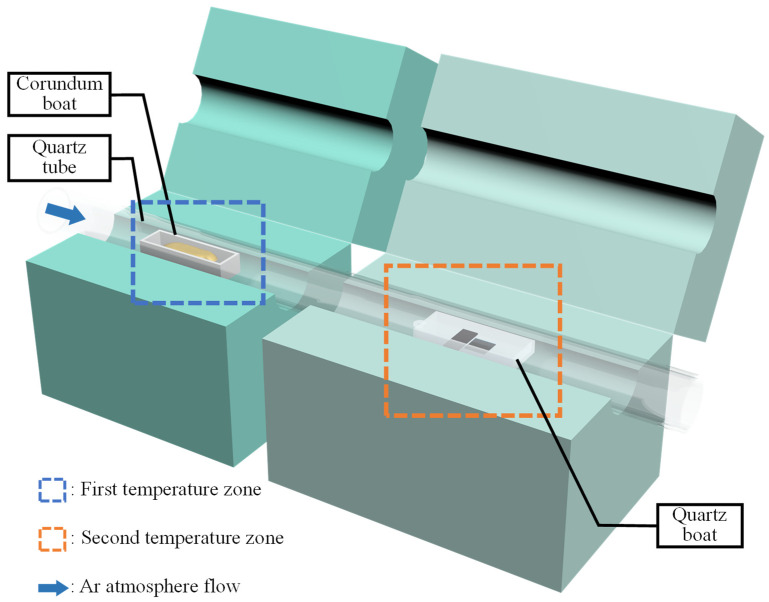
Schematic setup of chemical vapor deposition (CVD) system.

In this study, bilayer MoS_2_ was prepared with the assistance of NaCl, with S powder and MoO_3_ powder as an S source and Mo source, respectively, and soda-lime glass as the growth substrate. The schematic setup of the experiment is shown in Figure 1. This system adopted a double-temperature-zone tubular furnace for heating. The S source was placed in the corundum boat in the first temperature zone. The Mo source-growth booster and substrate were loaded into the quartz boat in the second temperature zone. In addition, to prevent the soda-lime glass substrate from melting and sticking to the quartz boat at high temperatures, Mo foil was needed under the soda-lime glass. After mixing NaCl solution and MoO_3_ powder on the silicon wafer and drying, the mixture was placed in the quartz boat, and then the Mo foil gasket and soda-lime glass substrate were placed on the right side. The quartz boat and the corundum boat containing S powder were placed in the two temperature zones of the CVD furnace, respectively. The temperatures of the S and NaCl-MoO_3_ sources were 200 °C and 890 °C, respectively. High-purity argon was loaded, with a flow rate of 20 sccm. During the growth, the CVD system was kept at atmospheric pressure for 10 min. After growth, the morphology and size of bilayer MoS_2_ were characterized by optical microscope and Raman spectroscopy. The sample images were collected using optical microscopy combined with CCD (OM, BX51M, OLYMPUS, Tokyo, Japan ) and an atomic force microscopy (Dimension Edge, Bruker, Karlsruhe, Germany). Raman spectra were analyzed by an embedded Raman spectrometer (K-Swns-523, ideaoptics, Shanghai, China). 

**Figure 2:**
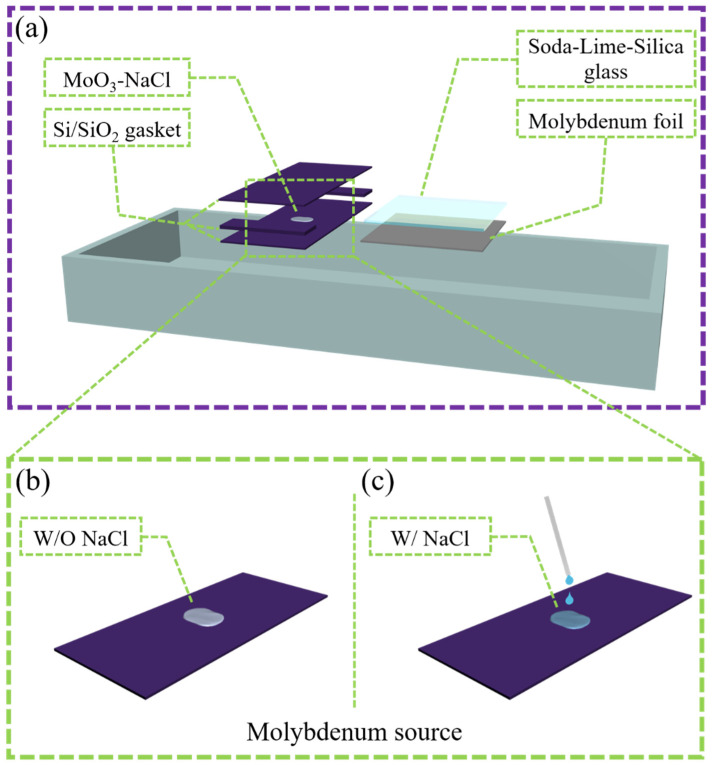
Schematic of Mo source and substrate placement: (**a**) Schematic map of Mo source and substrate placement; (**b**,**c**) schematic of Mo source with or without NaCl addition.

In this work, NaCl solution was directly mixed with MoO_3_ and dried as a Mo source for CVD-based growth of bilayer MoS_2_. The placement methods of the Mo source and substrate are presented in Figure 2. The Mo source was loaded into the quartz boat with SiO_2_/Si, which was covered with SiO_2_/Si cover plate to form a microcavity to prevent direct contact with S and poisoning during CVD. The soda-lime glass substrate was placed 3 mm to the right of the Mo source (at the downstream of the atmosphere flow), and the Mo foil was placed below to prevent it from sticking to the quartz boat after melting at high temperature.

## 3. Results and Discussion

### 3.1. NaCl-Assisted Growth of Bilayer MoS_2_

**Figure 3:**
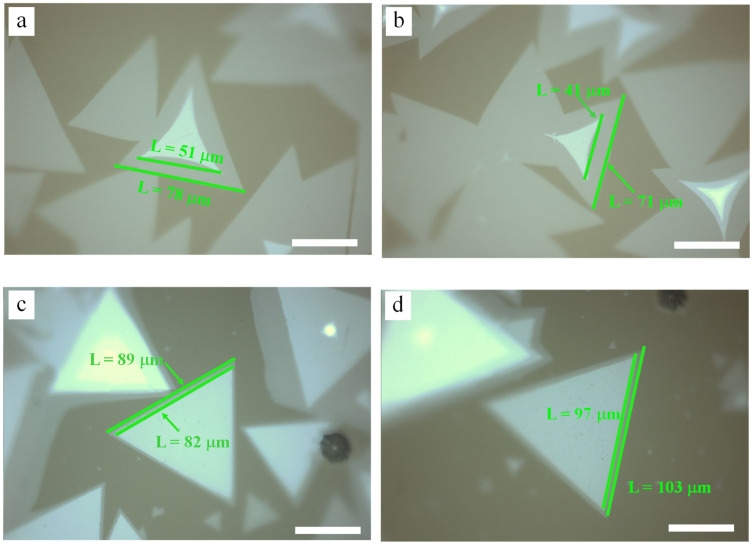
Comparison of growth results of NaCl-assisted bilayer MoS_2_. (**a**,**b**) Growth results without NaCl. (**c**,**d**) Growth results with NaCl. (**a**–**d**) The scale bars are 40 μm.

To explore the effect of NaCl on the growth of bilayer MoS_2_, a series of comparative experiments were carried out. First, with other growth conditions unchanged, the effects of NaCl assistance and non-NaCl assistance on the growth of bilayer MoS_2_ on the glass substrate were investigated. During the experiment, the temperature and mass of the S source were 200 °C and 1.4 g, the temperature and mass of the Mo source were 890 °C and 4 mg, and the growth pressure was 1000 mbar. In the NaCl group, the proportion of NaCl solution/MoO_3_ was 1 mL/300 mg, and the concentration of NaCl was 5 mol/L. After adding NaCl, drying was performed at 50 °C for 5 min for subsequent growth operations. The growth results were obtained through the use of an optical microscope and are shown in Figure 3. Through comparison, it was found that among the bilayer MoS_2_ obtained in the NaCl group, the crystal size and the proportion of the bilayer structure to monolayer structure increased significantly. Without NaCl addition, the proportion of the bilayer structure in the grown MoS_2_ crystals was about 40%, and the bilayer size was about 50 μm. With NaCl added, the proportion of bilayer structure in the MoS_2_ crystals was about 85%, and the bilayer size was about 100 μm. The comparison of these experimental results showed that alkaline metal halides could significantly promote bilayer crystal growth during the preparation of transition metal halides. The increased bilayer MoS_2_ domain size and proportion of bilayer structure has could contribute to the following two aspects: Firstly, MoO_3_ reacts with NaCl at a high temperature to produce an intermediate product (molybdenum dichloride dioxide, Cl_2_MoO_2_; melting point—184 °C), which can be evaporated rapidly compared with the high melting point of MoO_3_, reducing the temperature of the gas-phase reaction, and providing more Mo sources during the same period [44,48]. Additionally, Na in NaCl and melted soda-lime glass can be used as the nucleation initiation factor of MoS_2_, which can reduce the reaction barrier [37].

### 3.2. Effect of Mo Mass under NaCl Assistance

**Figure 4:**
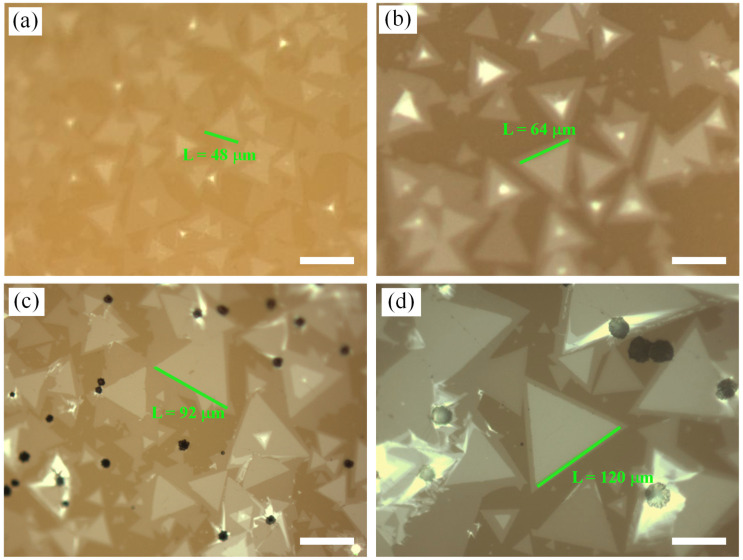
Growth results under different Mo sources masses assisted by NaCl: (**a**) 3 mg, (**b**) 3.5 mg, (**c**) 4 mg, and (**d**) 4.5 mg. (**a**–**d**) The scale bars are 60 μm.

A large number of studies have shown that the mass of precursors has an important effect on the growth of MoS_2_ [4,6,13,37]. Under NaCl-assisted growth, we studied the effect of changing Mo mass on the growth of bilayer MoS_2_. With NaCl/MoO_3_ = 1 mL/300 mg (NaCl concentration was 5 mol/L) and other process parameters unchanged (i.e., the temperature and mass of S source were 200 °C and 1.4 g; the temperature of Mo source was 890 °C; the growth pressure was 1000 mbar; and the growth time was 8 min), the effects of Mo sources with different masses (3 mg, 3.5 mg, 4 mg, and 4.5 mg) on the growth of bilayer MoS_2_ on the glass substrate were explored. The growth results were obtained through the use of an optical microscope and are shown in Figure 4. The morphologies of bilayer MoS_2_ in Figure 4a–d are consistent, presenting an equilateral triangle, with a high proportion of bilayer structure. Meanwhile, with the increase in Mo mass, the size of bilayer MoS_2_ domains also increased obviously, with the maximum size reaching 120 μm. This phenomenon is consistent with our previous work without NaCl assistance [13]. With the CVD process located in the mass transport limited region, higher precursor mass flux can contribute to a higher diffusion rate through boundary layer, resulting in overcoming the mass transport limit and promoting single domain growth [49,50].

### 3.3. Effect of Growth Pressure under NaCl Assistance

**Figure 5:**
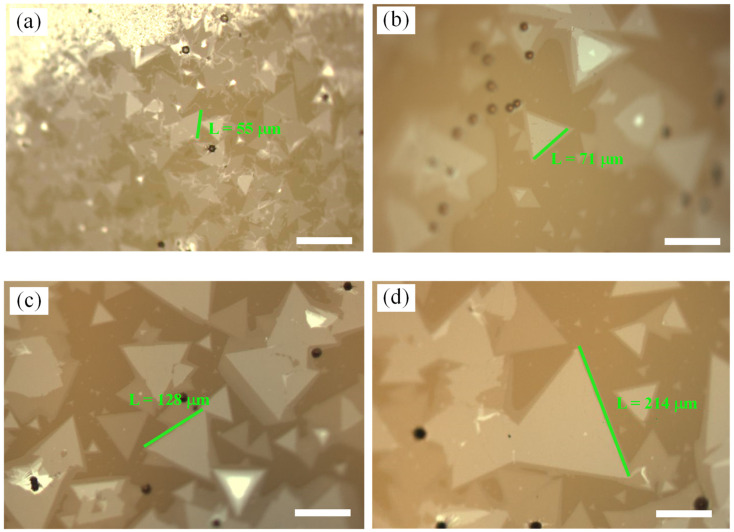
Growth results under different growth pressures assisted by NaCl. (**a**) The growth pressure is 1100 mbar. (**b**) The growth pressure is 1100 mbar. (**c**) The growth pressure is 900 mbar. (**d**) The growth pressure is 700 mbar. All scale bars are 100 μm.

In addition, with the assistance of NaCl, we further explored the effect of reaction pressure on the growth of bilayer MoS_2_ on the glass substrate. Similarly, with the mass ratio of NaCl/MoO_3_ and other growth conditions unchanged, different growth pressures (1100 mbar, 1000 mbar, 900 mbar, and 700 mbar) were used, and the growth results were obtained through the use of an optical microscope and are shown in Figure 5a–d. The size of bilayer MoS_2_ domains increased gradually with the gradual decrease in pressure, and the size of bilayer MoS_2_ single crystal growth at 700 mbar reached 214 μm, which is comparable to the largest bilayer MoS_2_ domain reported in literature [13,41]. The large bilayer MoS_2_ domain could be attributable to the assistance of NaCl and the low growth pressure. With the assistance of NaCl, the molybdenum oxychloride precursor with a low melting point produced volatilizes rapidly at high temperature, breaking the self-limiting growth of monolayer MoS_2_ [44], and then causing secondary nucleation and the synchronous growth of the bilayer structure. Moreover, under low pressure, the precursor diffused faster on the melted substrate, which contributed to the growth rate of bilayer structures closer to that of single-layer structures [37]. Therefore, the size of bilayer MoS_2_ could be greatly increased while maintaining a high proportion of the bilayer structure. 

**Figure 6:**
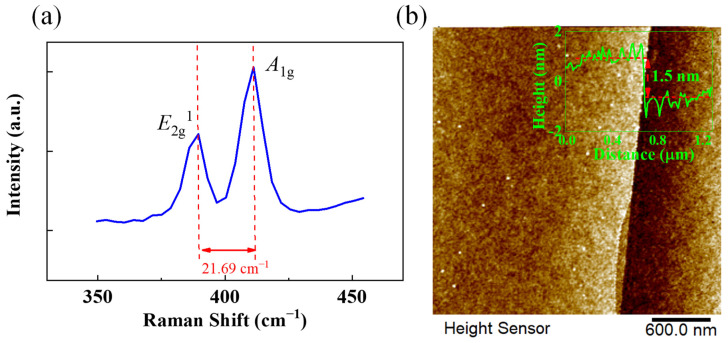
(**a**) Raman spectral of bilayer MoS_2_; (**b**) AFM image of bilayer MoS_2_.

Raman characterization is an important means to characterize the growth results of MoS_2_, which can reflect the layer number and quality of MoS_2_. In this study, the bilayer MoS_2_ crystals prepared under different pressures were characterized and analyzed by Raman spectroscopy. The Raman spectral characterization results are shown in Figure 6a. The characteristic peak *E*_2g_^1^ and *A*_1g_ of bilayer MoS_2_ crystals in the Raman spectral were located at 389.54 cm^−1^ and 411.23 cm^−1^, respectively, and the difference between the characteristic peaks was 21.69 cm^−1^. This result is consistent with the Raman characteristic peak position and difference of bilayer MoS_2_ in other studies, indicating that high-quality bilayer MoS_2_ crystals are obtained [51,52]. Furthermore, to demonstrate the bilayer nature of the MoS_2_ domains, Figure 6b displays the atomic force microscopy (AFM) images obtained from an edge of bilayer MoS_2_ domain. A thickness of 1.5 nm was demonstrated through the AFM characterization, which is consistent with the thickness of bilayer MoS_2_ in other works [13,53].

## 4. Conclusions

In summary, this study systematically investigates the NaCl-assisted growth of bilayer MoS_2_ on the soda-lime glass substrate. The results show that the introduction of NaCl plays a very important role in the growth of bilayer MoS_2_, and improves the proportion of bilayer structure and the size of bilayer MoS_2_ crystals. In addition, the effects of two important growth parameters, Mo mass and growth pressure, on the growth of bilayer MoS_2_ on the glass substrate under the assistance of NaCl are explored. The results reveal that, under certain conditions, the size of bilayer MoS_2_ crystals increases with the increase in Mo mass. Moreover, by changing growth pressure, the size of bilayer MoS_2_ crystals is significantly increased, and the domain size of bilayer MoS_2_ crystals is larger than 200 μm, with the bilayer structure accounting for about 85%. This study provides an interesting method worthy of further research for the growth of high-quality bilayer MoS_2_ and has significance for promoting the further large-scale low-cost synthesis and industrial application of two-dimensional MoS_2_.

## Data Availability

The data that support the findings of this study are available from the corresponding author upon reasonable request.
